# Small Bowel Herniation Into a Post-bariatric Pannus After Cesarean Delivery Managed With Single-Stage Panniculectomy and Rives-Stoppa Repair: A Case Report

**DOI:** 10.7759/cureus.112024

**Published:** 2026-07-03

**Authors:** Hannah Tan, Caleb W Brown, Phillip L Nichols, Derek C Wenger, Jeremy M Powers

**Affiliations:** 1 Surgery, Quillen College of Medicine, East Tennessee State University, Johnson City, USA; 2 Plastic Surgery, Quillen College of Medicine, East Tennessee State University, Johnson City, USA

**Keywords:** abdominal wall reconstruction, cesarean section, panniculectomy, post-bariatric surgery, retrorectus mesh, rives-stoppa repair, ventral hernia

## Abstract

Redundant pannus after massive weight loss can impair function and obscure abdominal wall pathology. Incisional hernia after cesarean delivery is uncommon, and extension of the small bowel into a post-bariatric pannus is particularly unusual. We report a 42-year-old woman with three prior cesarean deliveries and prior bariatric surgery who presented with a functionally limiting grade 3 pannus and a long-standing, nonreducible lower abdominal bulge. Computed tomography (CT) demonstrated a 12.3 cm × 5.6 cm low ventral incisional hernia with a 6.7 cm neck containing small bowel loops extending into the pendulous pannus. A single-stage operation involving plastic and general surgery was planned for combined panniculectomy and Rives-Stoppa repair. The patient underwent panniculectomy with umbilical sacrifice for exposure, bilateral retrorectus dissection with myofascial release, posterior sheath closure, and Rives-Stoppa repair using a 20 cm × 25 cm retrorectus mesh. She was discharged on postoperative day 1. A symptomatic retrorectus seroma developed within the first postoperative month and was successfully managed with image-guided percutaneous drainage; cultures were negative, and no recurrence or wound breakdown was present at three-month follow-up. This case highlights the value of multidisciplinary planning and carefully selected single-stage panniculectomy with retromuscular hernia repair for complex abdominal wall defects concealed by a post-bariatric pannus.

## Introduction

Massive weight loss, whether achieved through bariatric surgery or lifestyle modification, frequently results in redundant abdominal skin and subcutaneous tissue and the formation of a dependent pannus. This excess tissue may impair mobility, complicate hygiene, contribute to chronic intertriginous dermatitis, and reduce quality of life. Body-contouring procedures such as panniculectomy and abdominoplasty are therefore commonly performed after massive weight loss and may improve patient-reported physical function and psychosocial well-being [1,2]. Substantial abdominal soft-tissue redundancy may also complicate assessment of the underlying abdominal wall. In addition to addressing these functional concerns, panniculectomy may expose underlying abdominal wall pathology that is otherwise difficult to appreciate on physical examination in patients with substantial soft-tissue redundancy [3,4].

Incisional hernia is a well-established long-term complication of abdominal surgery, occurring through a fascial defect at or near the site of a previous abdominal wall incision. Although the incidence after cesarean delivery is lower than after many other abdominal operations, reported rates range from 0.0% to 5.6% [5]. Risk increases in patients with obesity, multiple prior operations, impaired wound healing, or chronically elevated intra-abdominal pressure [6]. Incisional hernias may remain asymptomatic for extended periods but can progressively enlarge and eventually present with pain, bowel involvement, or a risk of incarceration [6]. In patients with a large pannus or altered abdominal wall anatomy following massive weight loss, detection of these defects can be particularly challenging because redundant soft tissue may obscure abnormalities on physical examination [3,4,6].

Accurate preoperative assessment is therefore critical in patients with complex abdominal wall anatomy. Preoperative computed tomography (CT) evaluates ventral and incisional hernias, as it allows detailed visualization of abdominal wall layers, measurement of fascial defects, and identification of hernia contents [7]. CT imaging also assists surgeons in operative planning by defining dissection planes and reconstructive strategy prior to complex abdominal wall repair [8]. When repair is required for large ventral or incisional hernias, the Rives-Stoppa technique is a retromuscular repair in which mesh is positioned in the retrorectus/extraperitoneal plane, allowing broad mesh overlap while avoiding direct bowel-mesh contact [9].

Recognition of an occult abdominal wall defect during evaluation for panniculectomy is clinically important because it may significantly alter surgical planning and necessitate coordinated management between plastic and general surgeons. The purpose of this case report is to describe a low cesarean-associated incisional hernia containing small bowel that extended into a dependent post-bariatric pannus and was difficult to characterize on physical examination. We also highlight the role of CT imaging, multidisciplinary planning, and carefully selected single-stage panniculectomy with Rives-Stoppa retromuscular hernia repair.

This work was previously presented as a poster at the Tennessee Chapter of the American College of Surgeons Annual Meeting and Trauma Symposium in Buchanan, Tennessee, on August 10, 2025.

## Case presentation

A 42-year-old White woman was initially evaluated in January 2023 in plastic and general surgery clinics in Johnson City, Tennessee, USA, for panniculectomy and ventral hernia repair. Her history was notable for three prior cesarean deliveries and a Roux-en-Y gastric bypass three years earlier, resulting in a 42.6 kg weight loss (132.0 kg to 89.4 kg) and a reduction in body mass index from 44.3 kg/m² to 30.0 kg/m². Her medical history also included hypertension, transient ischemic attacks, upper-extremity deep venous thrombosis, anxiety, bradycardia previously evaluated by cardiology, daily aspirin use, and a remote history of tobacco use, having quit smoking more than one year before surgery. She first noticed a lower abdominal bulge shortly after her third cesarean delivery, approximately six years before presentation. Because the bulge began shortly after cesarean delivery and approximately three years before the Roux-en-Y gastric bypass, the defect was considered cesarean-associated rather than a complication of the bariatric procedure. The bulge remained stable for several years, and she did not undergo formal evaluation until after substantial weight loss, when the hernia became more clinically apparent. She denied nausea, vomiting, obstructive symptoms, or changes in bowel habits but reported intermittent abdominal fullness and functional limitation related to her pannus.

On physical examination, the abdomen was nondistended and nontender without rashes or skin lesions. She had a large grade 3 pannus, defined as a panniculus extending to cover the upper thigh [10]. With the patient in the supine position, visible peristaltic movements were observed through the overlying pannus, raising suspicion for a bowel-containing herniation. Further palpation was concerning for a large, nonreducible ventral hernia below the umbilicus (Figure 1).

**Figure 1:**
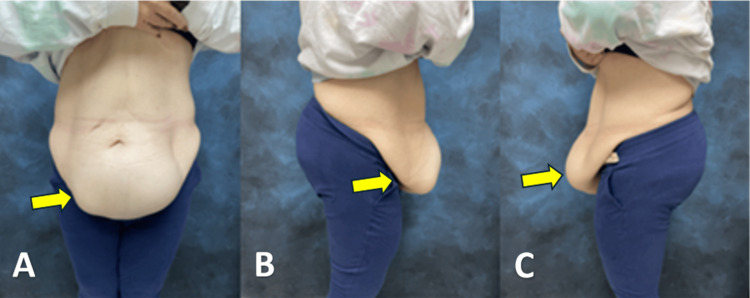
Preoperative clinical photographs (A) Anterior view demonstrating a large, pendulous post-bariatric pannus (yellow arrow). (B) Right lateral view demonstrating the dependent inferior extent of the pannus (yellow arrow). (C) Left lateral view demonstrating the pendulous pannus, which obscured the underlying hernia on clinical examination (yellow arrow).

CT of the abdomen and pelvis demonstrated a low ventral incisional hernia containing small bowel loops extending into the pendulous pannus (Figure 2).

**Figure 2:**
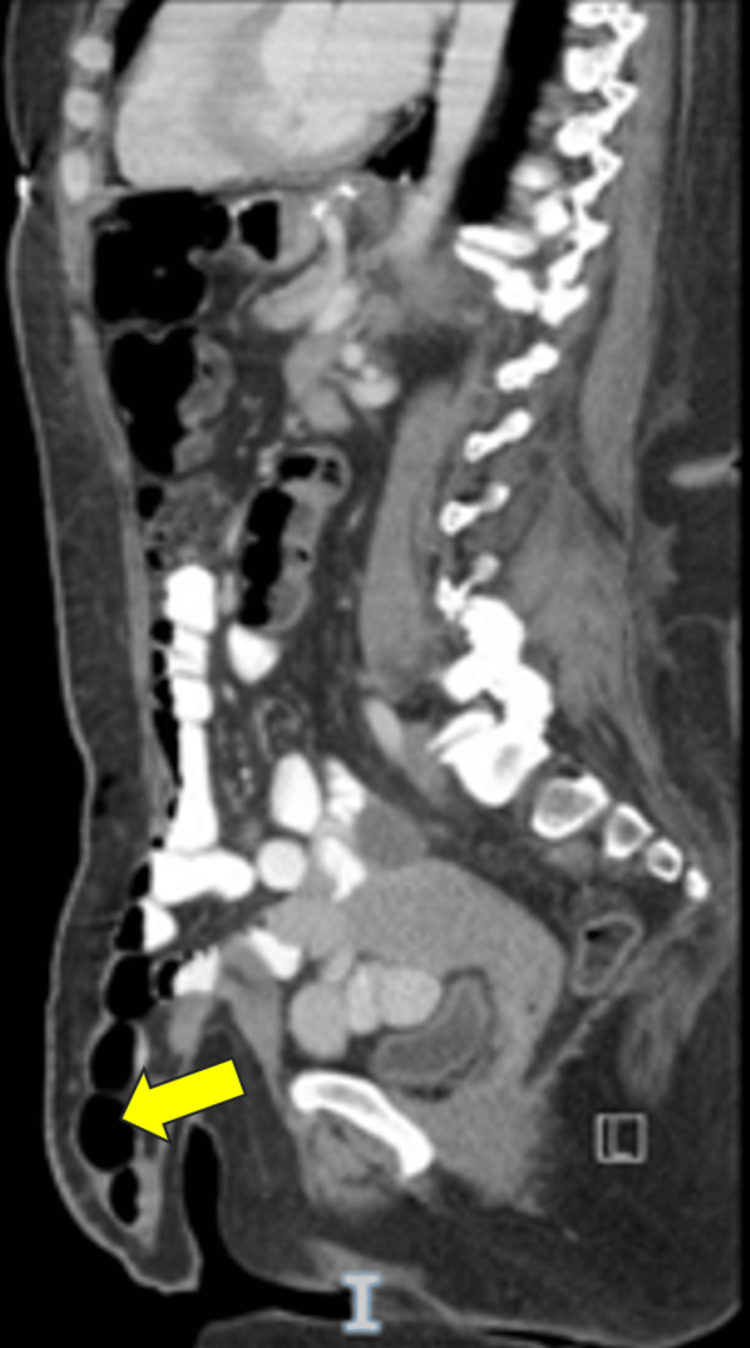
Sagittal CT of the abdomen and pelvis demonstrating a low anterior ventral hernia with herniation of small bowel loops into a pendulous post-bariatric pannus (yellow arrow). The hernia sac measured approximately 12.3 cm × 5.6 cm, and the neck was located at the level of the patient’s prior cesarean delivery site CT, computed tomography

The hernia sac measured 12.3 cm × 5.6 cm, and the neck measured 6.7 cm. Significant lower midline scarring related to prior cesarean deliveries was also present. Given the hernia size, soft-tissue redundancy, and anticipated need for wide exposure, a multidisciplinary plan was developed for single-stage panniculectomy and open Rives-Stoppa retrorectus ventral hernia repair. Preoperative cardiology evaluation deemed her an acceptable surgical candidate. She underwent combined panniculectomy and ventral hernia repair in May 2023.

Under general anesthesia, plastic surgery began with upper and lower transverse incisions based on a pinch test and dissected through the sub-Scarpa plane to the abdominal wall. The umbilicus was circumferentially dissected to the abdominal wall, the pannus was elevated from lateral to medial while preserving soft tissue over both anterior superior iliac spines, perforating vessels were controlled with hemoclips, and the pannus was excised en bloc. The umbilicus was sacrificed because of the anticipated disruption to its blood supply from the extensive retrorectus dissection.

General surgery then repaired the large lower abdominal wall defect at the prior cesarean scar. Extensive adhesions and scar tissue were carefully released, and the tissue planes behind the rectus muscles were developed to allow the abdominal wall to be brought back together in the midline. The posterior layer was closed. A trimmed 20 cm × 25 cm OviTex 1S permanent polymer-reinforced tissue matrix (TELA Bio, Malvern, PA, USA) was placed in the retromuscular space with adequate overlap, and the anterior fascial layer was closed over the mesh with additional reinforcing sutures.

Plastic surgery then re-entered the field for closure. Additional lateral and inferior skin was excised to minimize standing cone deformities. Progressive tension sutures were placed beneath the abdominal flap to reduce dead space and avoid surgical drains. Layered closure of Scarpa fascia, the deep subcutaneous tissue, and skin was completed, and a Prevena incisional negative-pressure dressing was applied.

The patient tolerated the procedure well and was discharged home on postoperative day (POD) 1 with pain controlled and the incisional negative-pressure dressing in place. During the first postoperative month, she developed abdominal fullness and focal pain. CT demonstrated a retrorectus fluid collection consistent with a seroma (Figure 3A).

**Figure 3:**
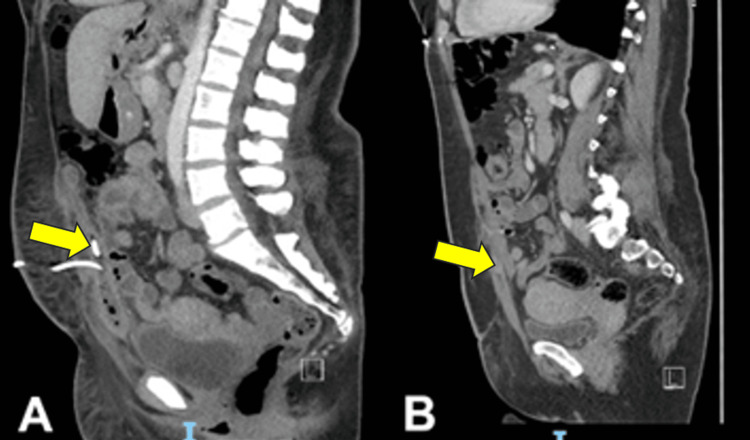
Postoperative CT (A) Sagittal CT on POD 35 demonstrating a retrorectus fluid collection consistent with a postoperative seroma (yellow arrow). A percutaneous drain is visible. (B) Follow-up sagittal CT three months postoperatively showing complete resolution of the seroma with no evidence of fluid reaccumulation or hernia recurrence (yellow arrow). The mesh remains in a stable position. POD, postoperative day; CT, computed tomography

The collection was successfully managed with image-guided percutaneous drainage, and cultures were negative. After drain removal, the lower abdominal incision remained clean, dry, and intact without breakdown. Follow-up CT at approximately three months showed complete resolution of the fluid collection, stable mesh position, and no recurrent hernia (Figure 3B). At the three-month outpatient follow-up, the panniculectomy closure remained well healed, and no recurrent abdominal wall fluid collection was palpable.

## Discussion

This case illustrates how post-bariatric soft-tissue redundancy can conceal clinically important abdominal wall pathology. Patients undergoing panniculectomy or abdominoplasty often report impaired hygiene, mobility, and quality of life preoperatively, and these outcomes often improve after body-contouring procedures [1,2]. At the same time, incisional hernia after cesarean delivery is uncommon but well recognized [5]. In our patient, the initial presentation was for panniculectomy consultation rather than hernia repair, but a long-standing cesarean-associated defect was difficult to characterize on physical examination because the large pannus engulfed the hernia sac contents and obscured the lower abdomen.

Although marked soft-tissue redundancy limited clinical characterization of the hernia, visible peristaltic movements through the overlying pannus while the patient was supine represented an unusual examination finding that raised suspicion for a bowel-containing herniation. Cross-sectional imaging was central to decision-making in our patient with complex post-bariatric anatomy. CT confirmed small bowel within the hernia sac and defined the 12.3 cm × 5.6 cm hernia sac, a 6.7-cm neck, and the relationship of the hernia to the dependent pannus. These findings informed operative planning, anticipated dissection planes, and the decision to pursue single-stage multidisciplinary repair rather than isolated panniculectomy or delayed hernia repair. In patients presenting for panniculectomy evaluation, preoperative CT should be strongly considered when examination is limited by body habitus or when hernia is suspected, because imaging may substantially alter the operative plan.

This case combines features of cesarean-associated incisional hernia, post-bariatric pannus, and complex abdominal wall reconstruction; accordingly, we discuss it in the context of the literature on concomitant panniculectomy with ventral hernia repair and retromuscular hernia repair. Literature on concomitant ventral hernia repair and panniculectomy remains mixed, but recent data support this approach in selected patients. Dias Rasador et al. found in a systematic review and meta-analysis that concurrent panniculectomy was associated with lower hernia recurrence but higher rates of surgical-site infection, surgical-site occurrence, reoperation, and longer length of stay [11]. In contrast, Elhage et al. reported in a propensity-matched cohort of complex abdominal wall reconstruction that concurrent panniculectomy was associated with higher wound occurrence but similar hernia recurrence and quality-of-life outcomes [12]. Gossett et al. likewise found in a large database review that concurrent panniculectomy was not associated with a significant increase in complications in selected patients [13]. In this case, a single-stage approach was favored to optimize operative exposure and eliminate the mechanical, downward traction of the heavy pannus on the fascial repair, mechanisms emphasized by Henriksen et al. and Giordano et al. [9,14]. Our patient had substantial weight loss and multidisciplinary preoperative assessment, along with optimization for nicotine cessation, which likely contributed to her favorable overall outcome.

For large midline ventral and incisional hernias, retromuscular mesh placement using the Rives-Stoppa technique is favored because it permits wide mesh overlap while avoiding direct contact between mesh and bowel [9]. These principles were relevant in this patient because CT demonstrated small bowel within the dependent hernia sac, and the intraoperative fascial defect measured 12 cm. Recent comparative work has also suggested shorter hospital stay and potentially fewer complications with extraperitoneal approaches relative to intraperitoneal onlay mesh repairs [9,15]. In our case, the open Rives-Stoppa repair complemented the wide exposure afforded by the panniculectomy and provided durable retromuscular reconstruction of the large fascial defect. The choice of a reinforced tissue matrix (OviTex 1S) in the retrorectus plane reflected a preference for structured biologic reinforcement in a high-risk surgical field undergoing simultaneous soft-tissue excision.

Despite the use of progressive-tension sutures and incisional negative-pressure wound therapy to minimize dead space, the patient’s postoperative course was complicated by a symptomatic retrorectus seroma. The seroma resolved after image-guided percutaneous drainage, and the patient maintained a well-healed wound and stable abdominal wall reconstruction at three-month follow-up, demonstrating that postoperative surgical-site occurrences can be managed successfully without compromising repair integrity.

## Conclusions

This case describes a rare presentation of a low incisional ventral hernia containing small bowel extending into a post-bariatric pannus after cesarean delivery. In a patient who initially presented for panniculectomy consultation, visible peristaltic movements through the overlying pannus and palpation of a possible hernia raised concern for a bowel-containing herniation. When physical examination is limited by a large pannus and hernia is suspected, preoperative CT should be strongly considered to define hernia contents, clarify the diagnosis, and guide operative planning, as imaging findings may substantially alter the surgical approach and identify the need for combined hernia repair. Here, a single-stage panniculectomy and Rives-Stoppa repair safely achieved simultaneous soft-tissue debulking and retromuscular abdominal wall reconstruction. While this report is limited by its single-patient design and a short three-month follow-up, it demonstrates that carefully selected patients with complex post-bariatric anatomy can benefit significantly from coordinated, single-stage plastic and general surgical management.

## References

[1] Rios-Diaz AJ, Morris MP, Elfanagely O (2022). Impact of panniculectomy and/or abdominoplasty on quality of life: a retrospective cohort analysis of patient-reported outcomes. Plast Reconstr Surg.

[2] Berkane Y, Saget F, Lupon E (2024). Abdominoplasty and lower body lift surgery improves the quality of life after massive weight loss: a prospective multicenter study. Plast Reconstr Surg.

[3] Baucom RB, Beck WC, Holzman MD, Sharp KW, Nealon WH, Poulose BK (2014). Prospective evaluation of surgeon physical examination for detection of incisional hernias. J Am Coll Surg.

[4] Kroese LF, Sneiders D, Kleinrensink GJ, Muysoms F, Lange JF (2018). Comparing different modalities for the diagnosis of incisional hernia: a systematic review. Hernia.

[5] Paulsen CB, Zetner D, Rosenberg J (2020). Incisional hernia after cesarean section: a systematic review. Eur J Obstet Gynecol Reprod Biol.

[6] Sanders DL, Pawlak MM, Simons MP (2023). Midline incisional hernia guidelines: the European Hernia Society. Br J Surg.

[7] Adams ST, Slade D, Shuttleworth P (2023). Reading a preoperative CT scan to guide complex abdominal wall reconstructive surgery. Hernia.

[8] Ghafoor S, Hoppe AT, Lange M (2024). Computed tomography for evaluation of abdominal wall hernias-what is the value of the Valsalva maneuver?. Hernia.

[9] Henriksen NA, Bougard H, Gonçalves MR (2025). Primary ventral and incisional hernias: comprehensive review. BJS Open.

[10] (2026). Practice Parameter for surgical treatment of skin redundancy for obese and massive weight loss patients. https://www.plasticsurgery.org/documents/health-policy/guidelines/guideline-2017-skin-redundancy.pdf.

[11] Dias Rasador AC, Marcolin P, da Silveira CA (2024). The impact of simultaneous panniculectomy in ventral hernia repair: a systematic review and meta-analysis. Hernia.

[12] Elhage SA, Marturano MN, Deerenberg EB (2021). Impact of panniculectomy in complex abdominal wall reconstruction: a propensity matched analysis in 624 patients. Surg Endosc.

[13] Gossett AG, Leavitt JD, Hooks WB III, Hope WW (2025). Outcomes after ventral hernia repair with concurrent panniculectomy: a large database review. J Am Coll Surg.

[14] Giordano S, Salval A, Oranges CM (2024). Concomitant panniculectomy in abdominal wall reconstruction: a narrative review focusing on obese patients. Clin Pract.

[15] Wieland L, Alfarawan F, Bockhorn M, El-Sourani N (2024). Comparison of eTEP and IPOM for ventral hernia surgery in the early postoperative period: a retrospective cohort study of a tertiary university centre. Hernia.

